# New ways of working to support sustainable disease elimination

**Published:** 2017-03-03

**Authors:** Geordie Woods, Yael Velleman, Virginia Sarah

**Affiliations:** 1 Technical Adviser Neglected Tropical Diseases, Sightsavers, New Orleans, USA.; 2 Senior Policy Analyst on Health, WaterAid, London, UK.; 3 Chair, International Coalition for Trachoma Control (ICTC), London, UK.

**How can we ensure that Neglected Tropical Diseases (NTDs) are not just eliminated, but eliminated once and for all?**

This article explores the key role that water, sanitation and hygiene (WASH) interventions can play and what partnerships, programs and policies can be adopted to help see the end of certain diseases for good.

When talking about the end goal for a number of NTDs, we use the term *elimination as a public health problem* instead of control. This requires disease prevalence to be reduced to below specific threshold levels so that transmission levels are sufficiently low for fixed health facilities to treat cases so that specific community outreach programs are not required.

This carries a risk of resurgence to public health problem levels if the conditions for transmission have not changed. For diseases in which access to water, and poor sanitation and hygiene (WASH) plays a fundamental role, undertaking efforts to improve these conditions will reduce the risk of resurgence and, ultimately, enhance the sustainability of elimination efforts.

The transmission of schistosomiasis, lymphatic filariasis and trachoma is closely linked to poor WASH conditions, yet programs often focus on medical interventions, particularly mass drug administration. In order to prevent the spread of these diseases, a greater focus on WASH services is needed to reach elimination goals faster, reduce competition for resources and increase the value of programs in the eyes of the public and politicians by offering other health and non health related benefits.

This approach is being championed at the highest level. In August 2015, the World Health Organization (WHO) unveiled a global strategy and action plan to better integrate WASH services with public health interventions to accelerate progress in eliminating and eradicating NTDs by 2020. The emphasis is further stated in the standard operating procedures developed by WHO for validation or elimination of trachoma as a public health problem. The procedures require that gains against disease are sustained in the absence of antibiotic pressure and that evidence that environmental and behavioural conditions for transmission have been addressed. This provides added incentive for trachoma partners to work with WASH, education and other stakeholders.

While efforts have been made over the years by those working on NTDs to engage with agencies that deliver WASH services, NTD and WASH programs have tended to work separately. This has led to concerns over both the sustainability of achievements made through mass drug administration and over the lack of targeting of WASH services to endemic communities, which are almost always the communities most in need of those services.

**Find out more www.trachomacoalition.org/sustainability**

This challenge has been picked up within trachoma elimination efforts, predominantly through the two large-scale programs funded by the UK Department for International Development (DFID) and The Queen Elizabeth Diamond Jubilee Trust (The Trust). Planning workshops in seven countries aimed specifically at trachoma brought entirely new groups together, including different sectors of government (WASH, Education, Health) as well as NGOs and academics. This allowed for real-time development and testing of innovative planning tools. To enhance this collaboration and ensure lessons were captured and shared, in 2015 ICTC released *All you need for F&E - a toolkit for planning and partnering*, a resource aiming to strengthen coordination and maximise impacts in the field by supporting program managers working on trachoma to engage stakeholders from other/dependent sectors.

**Figure:**
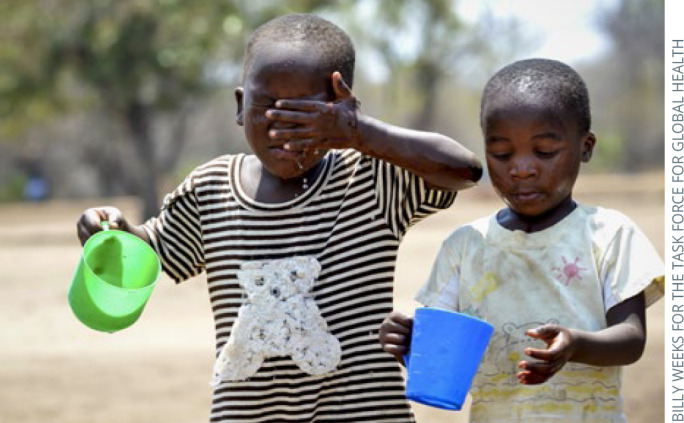
Good hygiene and access to water is essential for ensuring clean faces and reducing transmission, an essential strand of the strategy to eliminate trachoma. MALAWI

Another initiative will enhance joint WASH and NTD monitoring processes. An extensive consultation with NTD and WASH experts has resulted in an agreed set of core indicators to be applied at program level. By sharing goals and indicators, the common ground between partners is made explicit, making collaboration easier.

At the country level, Zambia and Kenya are providing us with some concrete examples of how data sharing can support joined-up working. In Zambia, a DFID supported trachoma elimination program has built upon an already successful monitoring platform to track the WASH-related elements of trachoma interventions in rural communities and monitor facial cleanliness and environmental improvement. In Kenya, a Trust supported trachoma elimination program in 12 counties has sourced demographic data from community health unit chalkboards, area and sub-county records (verified by education and health departments) to inform tailored planning for WASH components of SAFE, the trachoma intervention strategy, that would not have been possible using analysis of national-level data.

Ensuring the sustainability of elimination efforts is possible. There are practical examples to support the sustainable elimination of some of the world's most disabling and neglected diseases. We look forward to seeing these efforts scaled up and adopted by more partners working on trachoma and other NTDs.

